# The efficacy of first-line regimens for *Helicobacter pylori* eradication in different continents: A systematic review and network meta-analysis protocol

**DOI:** 10.1097/MD.0000000000013682

**Published:** 2018-12-14

**Authors:** Mohammad Zamani, Vahid Zamani, Mohammad H. Derakhshan, Javad Shokri-Shirvani

**Affiliations:** a Student Research Committee, School of Medicine; b Cancer Research Center, Health Research Institute; c Vice-Chancellery for Health, Babol University of Medical Sciences, Babol, Iran; d College of Medical, Veterinary & Life Sciences, University of Glasgow, Glasgow, UK; e Department of Internal Medicine, Rohani Hospital, Babol University of Medical Sciences, Babol, Iran.

**Keywords:** eradication, *Helicobacter pylori*, network meta-analysis, systematic review

## Abstract

**Background:**

Striking prevalence of *Helicobacter pylori* infection has been convoluted with considerable resistance to various antibiotics worldwide. Although many eradication regimens have been introduced as the first-line therapies against *H pylori*, lack of appropriate multiple comparison studies makes hard to implement such results to the clinical practice. This project attempts to utilize a comprehensive network meta-analysis to pool the results of clinical trial comparing various first-line eradication therapies simultaneously in different continents.

**Methods:**

We will include all randomized controlled trials assessing the first-line regimens for treatment of *H pylori* published in last 10 years. We will search the databases of PubMed, EMBASE, Scopus, Web of Science and Cochrane Central Register of Controlled Trials, International Standard Randomised Controlled Trial Number registry, World Health Organisation International Clinical Trials Registry Platform, and ClinicalTrials.gov for randomized controlled trials published since January 2009 without language limitation. The primary and secondary outcomes will be *H pylori* eradication rate and adverse events, respectively. Subgroup analyses will be conducted for different continents. Two reviewers will independently contribute in study selection and data extraction. For evaluating quality of studies, Cochrane Collaboration tool score will be used. We will conduct network meta-analysis for treatment comparisons using STATA software version 13.

**Results:**

These findings will be submitted to a peer-reviewed journal for publication.

**Conclusion:**

Our results will provide the guidance for clinicians in deferent regions to select the best possible therapeutic regimen for treatment of *H pylori* infected patients.

**Registration number:**

This systematic review and network meta-analysis has been registered in the PROSPERO International Prospective Register of Systematic Reviews, with registration number CRD42017077061.

## 1 Introduction

### 1.1 Rationale

*Helicobacter pylori* (*H pylori*) is a gram-negative flagellated microaerophilic spiral-shaped bacterium and is the important cause of gastric diseases, such as peptic ulcer disease, chronic gastritis, gastric adenocarcinoma, and mucosa associated lymphoid tissue lymphoma.^[1,2]^ An overall prevalence of 44% makes it the most common infection in the world, with considerable variation form 26% in Northern America to 59% in Latin America.^[3]^

According to the Kyoto global consensus, almost all *H pylori* infected persons need to be offered eradication therapy.^[4]^ Patients with gastric or duodenal ulcers, those with gastric precancerous lesions, and patients undergoing long-term therapy with proton pump inhibitors (PPIs) are typical examples of groups with clear indications of *H pylori* eradication.^[5]^

Once the eradication is necessary, this is performed by prescription of various combinations of antibiotics plus a PPI. Until recently, the well-established combinations for the first-line treatment is suggested by the Maastricht V consensus,^[5]^ which was a standard triple therapy consisted of clarithromycin and amoxicillin/metronidazole plus one proton pump inhibitor. Not surprisingly, its efficacy has been declined over the past years, much lower than recommended optimal success rate.^[6,7]^ In most world regions, the eradication rate of the triple therapy is <80%.^[8]^ The main reason for this failing is attributed to clarithromycin resistance.^[9]^ A recent review alarmed an increasing trend in primary *H pylori* antibiotic resistance in the world.^[10]^ Therefore, it is necessary to determine the therapeutic status of *H pylori* infection globally. Until now, two network meta-analyses have been published to assess this issue,^[11,12]^ but there are some limitations about them. For instance, the searched databases were limited to maximum 3 websites. Also, they did not evaluate the efficacy of the regimens in different continents, considering that each continent has its own socioeconomic status and health policy.

### 1.2 Objectives

Our primary objective is to comprehensively evaluate and compare the eradication rates of various pharmacological *H pylori* treatment regimens together in different continents. The comparison will include direct and indirect comparison in the absence of direct evidence of superiority of one eradication regimen to another, using network analyses. The second objective is to determine the rates of adverse effects of different regimens leading to treatment discontinuation if applicable.

## 2 Methods

### 2.1 Overview

**Figure 1:**
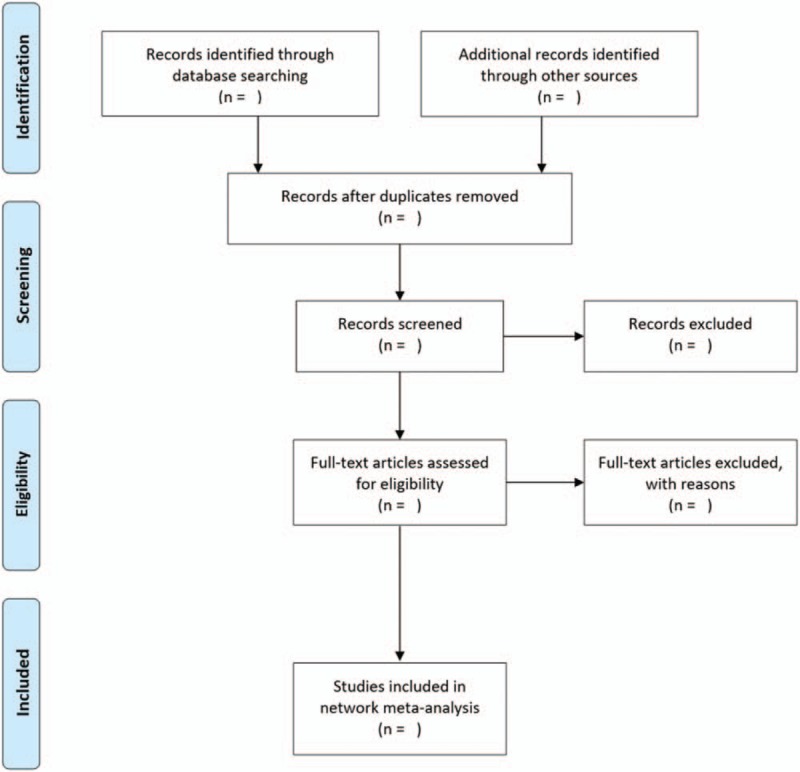
PRISMA flow diagram. PRISMA = Preferred Reporting Items for Systematic reviews and Meta-Analysis for Protocols.

This systematic review protocol is registered in the PROSPERO International Prospective Register of Systematic Reviews, with registration number of CRD42017077061. For preparation and reporting the present protocol, we used the Preferred Reporting Items for Systematic reviews and Meta-Analysis for Protocols 2015 (PRISMA-P 2015).^[13]^ The information of different steps of this systematic review will be indicated in the PRISMA flow diagram (Fig. 1).^[14]^ Also, our further results will be presented according to the guidelines of the PRISMA extension statement for network meta-analyses.^[15]^

### 2.2 Eligibility criteria

#### 2.2.1 Participants and settings

We will include all original randomized controlled trials (RCTs) which recruited patients who were:

(a) Diagnosed with *H pylori* infection regardless of the reason for eradication (although reasons will be reported as additional information).
(b) Diagnosis will be based on one positive results on one of established techniques including Rapid Urease Test, Histology (with specific staining), Urease Breath Test (C^13^ or C^14^), or any molecular methods. Serological measurements are not acceptable.
(c) All recruited patients in the original RCTs are adults (based on definition in the original studies).
(d) All patients have their first attempt of eradication therapy.

The studies conducted on the patients with comorbidity (such as renal failure) will be also excluded.

#### 2.2.2 Interventions

All interventions in the clinical trials concerning *H pylori* infection with following conditions will be included in network analysis:

(a) Randomized controlled, either blinded or open
(b) RCTs with ≥2 therapeutic arms
(c) RCT groups are defined by their type, duration, and characteristic component
(d) Acceptable types are: (A) Triple Combination, (B) Quadruple Combination (such as bismuth-based and concomitant regimens), (C) Sequential, and (D) hybrid
(e) Possible durations are: (A) 7 days, (B) 10 days, (C) 14 days.
(f) Characteristic components are: (A) Clarithromycin, (B) Quinolone, (C) Bismuth, (D) Furazolidone, (E) Metronidazole

#### 2.2.3 Comparators

The main comparators are eradication therapy combinations, which will not include H2 blockers, probiotics, or herbal compounds. All RCTs comparing ≥2 interventions within the same studies are acceptable.

#### 2.2.4 Outcomes

We will only include a study if it reports the eradication rate (%) in form of intention to treat (ITT) (primary outcome). In the absence of ITT record, we will contact the corresponding author. Secondary outcomes will be adverse effects that are responsible for treatment discontinuation.

#### 2.2.5 Language and time frame

We will include studies with no language restriction. For non-English articles, Google Translate will be used. Considering significant progress in the eradication regimens, the publication year will be limited to last 10 years commencing first day of January 2009.

### 2.3 Information sources and search strategy

We will search the databases of PubMed, EMBASE, Web of Science, Scopus, and Cochrane Central Register of Controlled Trials (CENTRAL) for RCTs published since January 2009 without language limitation. We will also search the International Standard Randomised Controlled Trial Number registry, the World Health Organisation International Clinical Trials Registry Platform and the ClinicalTrials.gov to ensure identification of all eligible studies.

Hand search of the references of the retrieved studies and the relevant review articles are additional source of potential studies to retrieve any missing articles during computerised search.

#### 2.3.1 Box Search strategy for PubMed

For this systematic review, we will use keywords

#1: “*Helicobacter pylori*”
#2: “*H pylori*”
#3: “Helicobacter”
#4: #1 OR #2 OR #3
#5: “treatment”
#6: “treatments”
#7: “eradication”
#8: “eradications”
#9: “regimen”
#10: “regimens”
#11: “therapy”
#12: “therapies”
#13: #5 OR #6 OR #7 OR #8 OR #9 OR #10 OR #11 OR #12
#14: #4 AND #13.

The search will be limited to Title/Abstract in PubMed, EMBASE, and Scopus databases. It will be limited to Topic in the Web of Science. The search limitation will be applied to Title/Abstract/Keywords in CENTRAL.

For searching in the registries, the “Condition” will be defined as “*Helicobacter pylori*” OR “*H pylori*”.

### 2.4 Study selection

Endnote software will be used for managing references obtained through the databases search. A data extraction form will be developed and the gathered citations will be assessed independently by two reviewers (MZ and VA). About applying eligibility criteria, the 2 reviewers will primarily practice on a small subset and check the agreement level, trying to have >90% agreement (by Kappa). When reached to 90% agreement, they will start the main work. If there was any discrepancy, it will be resolved through a consensus between the authors.

### 2.5 Data extraction

After inclusion of the eligible studies, the following essential data will be included, but not limited to: author's name, publication year, country, RCT type (open, single blinded, double blinded, triple blinded), diagnostic method (before treatment), diagnostic method (after treatment), intervention arms, number of subjects in each arm, ITT (%), and discontinuation cause. Full-texts and/or any necessary information which were not available will be asked the authors by email.

### 2.6 Outcome measures

Eradication rate (or success rate) is the primary outcome in this systematic review and network analysis. It is defined as negative results in all tests done for diagnosis of *H pylori* infection after treatment and will be presented as a percentage. Each interventional arm will have one eradiation rate from each study. Adherence to intervention, adverse events that lead to discontinuation of intervention are secondary outcomes, which might not available for all studies.

### 2.7 Assessment of risk of bias

We will use the Cochrane revised tool for Risk of Bias (RoB V.2.0)^[16]^ to examine the all selected studies for bias individually concerning: bias arising from randomization process, deviation from intended interventions, missing outcome data, bias in outcome measurement, and bias in selection of the reported result. The tool will calculate an overall score of bias based on all above 5 items. Again, 2 reviewers will do this assessment independently and in case of disagreement, this will be solved through discussion with an additional reviewer.

### 2.8 Statistical analysis

#### 2.8.1 Conventional meta-analysis

We will start with pairwise meta-analysis to compare eradication rates head to head. Considering 48 combinations theoretically this will produce many comparisons, but there are far less available combinations in published RCTs. In addition, we need at least 2 RCTs for each comparison to conduct a meta-analysis. Pooled eradication rates will be estimated using random or fixed effect methods where appropriate. To examine the heterogeneity, *Q* test, and I^2^ statistics will be reported. Sensitivity analyses are predicted to perform in case of significant heterogeneity indicated by *I*^2^ > 50%. Funnel charts will be created to illustrate the risk of publication bias. Begg and Egger tests will be also used for assessing the publication bias. The main analyses will be performed using STATA v13 (StataCorp, College Station, TX) statistical package.

#### 2.8.2 Network analysis

To compare eradication rates together, we will perform network meta-analysis. The method of choice is Markov chains Monte Carlo within Bayesian framework. In this method, we are able to run multiple chains in same time with a minimum of 10,000 simulations in each chain. In order to decide between random effect and fixed effect model, we will run a preliminary analysis using each method to have an estimate for deviance information criteria (DIC). The method with smaller DIC will be preferred for the final analysis. To evaluate the magnitude of the global heterogeneity, we will use *I*^2^ statistics for network analysis.

It is wise to assess the effects of various factors on eradications rates achieved by an individual combination, that is, geographical location, sex, and sample size. When such informations are available, we will perform meta-regression analysis. Subgroup analyses will be justified if results of meta-regression revealed the presence of a significant confounder. We will do subgroup analyses to investigate the rank ordering of the regimens based on the following items if possible: different continents; countries with high (>15%) and low (<15%) clarithromycin and metronidazole resistance; publication date; countries with high and low incidence of gastric cancer. To determine the prevalence of *H pylori* resistance to clarithromycin and metronidazole, we will conduct a literature review on the previous publications. For example, regarding the rates of antibiotic resistance in Asia, we will use the results presented by the recent meta-analysis on Asian countries^[17]^. High and low incidence of gastric cancer will be defined as rates of <15 and >15 per 100,000 population.^[18]^

## 3 Discussion

The treatment of *H pylori* infection is a challenge for physicians and health professionals due to increasing rates of antibiotic resistance in the world. This problem caused the researchers to perform RCTs to assess different types of therapeutic regimens in *H pylori* infected patients. Many RCTs published over the last years in this regard, and therefore, it is necessary to summarize those data and provide a reliable evidence to show which treatment is the best choice for eradication of the infection in these patients. Recently, 2 network meta-analyses have been published about this subject, but they faced some limitations which we plan to overcome by expanding the databases to be searched, evaluating the success of treatments in different regions of the world, and assessing therapeutic regimens not included in the 2 previous systematic reviews (in particular, furazolidone-based regimens). We will also perform a subgroup analysis to investigate the rank order of the regimens by countries with high and low incidence of gastric cancer, which is a new approach. We believe our results will be beneficial for clinicians to select the most appropriate regimen for treatment of *H pylori* infection based on region where the patients are treated.

## Acknowledgment

The authors are thankful to Dr. Reza Alizadeh-Navaei, Assistant Professor at Gastrointestinal Cancer Research Center of Mazandaran University of Medical Sciences, and to Dr. Abbas Ali Keshtkar, Assistant Professor at School of Public Health of Tehran University of Medical Sciences, for their advising on our study.

## Author contributions

MZ and JSS contributed in study design. MHD contributed in advising on study design. MZ and VZ contributed in writing the protocol draft. MHD and JSS contributed in revising the manuscript. All authors have read and approved the final manuscript.

**Conceptualization:** Javad Shokri-Shirvani.

**Formal analysis:** Mohammad H. Derakhshan.

**Investigation:** Mohammad Zamani.

**Validation:** Mohammad Zamani, Vahid Zamani, Javad Shokri-Shirvani.

**Writing – original draft:** Mohammad Zamani, Vahid Zamani.

**Writing – review & editing:** Mohammad H. Derakhshan, Javad Shokri-Shirvani.

Mohammad Zamani orcid: 0000-0003-1916-3873.

Vahid Zamani orcid: 0000-0001-7485-1842.

Mohammad H. Derakhshan orcid: 0000-0002-2549-7100.

Javad Shokri-Shirvani orcid: 0000-0002-2090-2234.
